# The rates of quinolone, trimethoprim/sulfamethoxazole and aminoglycoside resistance among Enterobacteriaceae isolated from urinary tract infections in Azerbaijan, Iran

**DOI:** 10.3205/dgkh000313

**Published:** 2018-08-16

**Authors:** Mina Yekani, Hossein Bannazadeh Baghi, Fatemeh Yeganeh Sefidan, Robab Azargun, Mohammad Yousef Memar, Reza Ghotaslou

**Affiliations:** 1 Immunology Research Center, Tabriz University of Medical Sciences, Tabriz, Iran; 2 Microbiology Department, School of Medicine, Tabriz University of Medical Sciences, Tabriz, Iran; 3 Infectious and Tropical Research Center, Tabriz University of Medical Sciences, Tabriz, Iran

**Keywords:** aminoglycosides, Enterobacteriaceae, quinolones, urinary tract infections, resistance

## Abstract

**Aim:** Antibiotic susceptibility patterns help to select appropriate empirical treatments of urinary tract infections (UTIs). This study aimed to investigate antibiotic resistance among Enterobacteriaceae isolated from UTIs in Azerbaijan, Iran.

**Methods:** This study was carried out during 2016 in hospitals located in Tabriz, Urmia, and Khoy. Midstream urine specimens were cultured and identified by the standard methods. Susceptibility testing was carried out using the disk diffusion agar method for cefotaxime, ceftazidime, ceftriaxone, cefoxitin, imipenem, meropenem, ertapenem, cefepime, ampicillin, cefazolin, cefuroxime, aztreonam, nitrofurantoin, and fosfomycin and the agar dilution method for MIC determination of aminoglycosides, quinolones, sulfamethoxazole, and trimethoprim.

**Results:** A total of 219 non-duplicated Enterobacteriaceae were isolated from UTIs. According to the agar dilution assay, the following resistance rates were determined: trimethoprim/sulfamethoxazole (co-trimoxazole) 69.8%, nalidixic acid 68.9%, ciprofloxacin 66.2%, levofloxacin 58.5%, tobramycin 47.9%, kanamycin 39.3%, gentamicin 27.8%, and amikacin 5.5%. High levels of resistance were observed to trimethoprim (78.5%), sulfamethoxazole (88.1%), ampicillin (86.3%), and cephazoline (79.4%).

**Conclusion:** The most effective agents against Enterobacteriaceae were fosfomycin, carbapenems, and amikacin. Quinolones, trimethoprim and sulfamethoxazole are not appropriate for empirical therapy due to high levels of resistance. Amikacin is more effective among aminoglycosides and may be more effective, in complicated cases, when used in combination with fosfomycin and carbapenems.

## Zusammenfassung

**Zielsetzung:** Antibiotika-Suszeptibilitätsmuster unterstützen die Auswahl von Antibiotika zur empirischen Behandlung von Harnweginfektionen (HWI). Daher sollte die Antibiotikaresistenz von bei Harnweginfektionen in Azerbaijan isolierten Enterobacteriaceae untersucht werden.

**Methode:** Die Studie wurde in 2016 in Krankenhäusern in Tabriz, Urmia und Khoy durchgeführt. Mittelstrahlurin wurde mittels üblicher Technik kultiviert. Die Resistenztestung wurde für Cefotaxim, Ceftazidim, Ceftriaxon, Cefoxitin, Imipenem, Meropenem, Ertapenem, Cefepim, Ampicillin, Cefazolin, Cefuroxime, Aztreonam, Nitrofurantoin und Fosfomycin mit dem Plättchentest, für Aminoglycoside, Chinolone, Sulfamethoxazol und Trimethoprim im Agardiffusionstest mittels Bestimmung der MHK durchgeführt.

**Ergebnisse:** Bei 219 verschiedenen Enterobacteriaceae spp. wurden folgende Resistenzraten ermittelt: Trimethoprim/Sulfamethoxazol (Cotrimoxazol) 69,8%, Nalidixinsäure 68,9%, Ciprofloxacin 66,2%, Levofloxacin 58,5%, Tobramycin 47,9%, Kanamycin 39,3%, Gentamicin 27,8% und Amikacin 5,5%. Hohe Resistenzraten wurden bei Sulfamethoxazol 88,1%, Ampicillin 86,3%, Cephazolin 79,4% und Trimethoprim 78,5% beobachtet.

**Schlussfolgerungen:** Am wirksamsten erwiesen sich gegen Enterobacteriaceae spp. Fosfomycin, Carbapeneme und Amikacin. Chinolone, Trimethoprim and Sulfamethoxazol sind nicht geeignet zur empirischen Therapie. Unter den Aminoglycosiden ist Amikacin wirksamer und in Kombination mit Fosfomycin und Carbapenemen bei schweren Verlaufsformen möglicherweise noch wirksamer.

## 1 Introduction

Urinary tract infections (UTIs) are one of the most frequent infections in humans and manifest in forms such as cystitis and pyelonephritis [1]. These infections are a common cause for hospitalization and are also frequently acquired in hospitalized patients suffering from other diseases [2], [3]. Enterobacteriaceae, including *Escherichia coli*, *Klebsiella pneumoniae/oxytoca*, and *Proteus mirabilis*, are the pathogens most frequently isolated from UTIs [4]. Asymptomatic bacteriuria occurs in 1–2% of school-aged girls and 5% of all women; however, it is uncommon in males. The frequency increases with age. UTIs are found in about 40% of all females during their lives and the probability of relapse is 30%.The majority of UTI cases are less severe, but some forms can cause renal failure and life-threatening sepsis [5]. 

Empirical therapy is guided by awareness of the etiology of UTIs and the outbreak of antibiotic resistance. Antibiotic resistance patterns vary in different hospitals, cities, and nations. Globally, resistance to generally administrated parenteral and oral antibacterial agents such as aminoglycosides, third-generation cephalosporins, carbapenems, and beta-lactam/beta-lactamase inhibitor combinations, sulfamethoxazole, trimethoprim, and trimethoprim/sulfamethoxazole (co-trimoxazole) is reported [2], [6], [7]. UTIs caused by resistant pathogens have a longer time for symptom resolution and higher re-consultation rates and more frequently require multiple courses of antibiotics therapy [5]. The antibiotic susceptibility patterns conferred by regional laboratories usually help to select the empirical therapy of UTIs. Commonly, antimicrobial therapy is initiated before susceptibility testing, which may lead to the overuse of antibiotics [8]. Identification of the UTI pathogens and knowledge of resistance patterns to commonly prescribed antimicrobial drugs in clinical settings is fundamental and helpful in increasing the influence of empirical therapy [9]. This study aimed to investigate aminoglycoside, quinolone and antimetabolite efficacy for treatment of Enterobacteriaceae isolates from UTIs in Azerbaijan, Iran. 

## 2 Materials and methods

### 2.1 Patients

This study was carried out at hospitals in Tabriz, Urmia, and Khoy during 2016 in patients with positive urine cultures. The size of the population studied was 219 patients. The sample size calculation was carried out according to the formula [Z^2^*(p)*(1–p)\d^2^] (Z=1.96 for 95% confidence level, p= expected proportion in the population expressed as a decimal, (7%), d= absolute error or precision, expressed as a decimal (4%)). Patients were selected according to mid-stream urine cultures sent to the microbiology laboratory. Patient consent forms were prepared and handed out to patients before specimen collection. The study was permitted by the Ethics Commission of Tabriz University of Medical Sciences (IR.TBZMED.REC.1395.847).

### 2.2 Bacterial isolates

One calibrated loopful of urine samples was inoculated on sheep blood agar and MacConkey agar plates. These plates were incubated aerobically at 37°C for 24 h. Only the samples that had positive urine cultures with considerable bacterial growth ≥10^5^ CFUs/mL were included. The bacterial isolates were identified by Gram-staining, oxidase, indole, urease, lysine decarboxylase, arginine dihydrolase, ornithine decarboxylase, acid produced from lactose, and phenylalanine deaminase tests, and culturing in triple sugar iron medium (TSI), Simmons’ citrate agar, SIM medium (Sulfide hydrogen, Iodole, Motility), Methyl Red, and Voges-Proskauer (MR-VP) [10].

### 2.3 Antibiotic susceptibility testing

#### Disk diffusion assay

The test was performed on Mueller-Hinton agar according to the Clinical and Laboratory Standards Institute (CLSI) guidelines. The antibiotic disks used (MAST diagnosis, England) included cefotaxime (30 µg), ceftazidime (30 µg), ceftriaxone (30 µg), cefoxitin (30 µg), imipenem (10 µg), meropenem (10 µg), ertapenem (10 µg), cefepime (30 µg), ampicillin (10 µg), cefazolin (30 µg), cefuroxime (30 µg), aztreonam (30 µg), nitrofurantoin (300 µg), and fosfomycin (200 µg). The colony suspension, equivalent to a 0.5 McFarland standard, was incubated on the Mueller-Hinton agar. After placing the disks on the agar surface, the plates were incubated aerobically at 37°C in ambient air for 18 hours. The results of the disk diffusion method were controlled using the American Type Culture Collection quality control strain *Escherichia coli* ATCC 25922 [11].

#### MIC determination

The minimum inhibitory concentration (MIC) of gentamicin, amikacin, kanamycin, tobramycin, ciprofloxacin, levofloxacin, nalidixic acid, trimethoprim, sulfamethoxazole, and trimethoprim/sulfamethoxazole was determined by the agar dilution method on Mueller-Hinton agar. The plates were incubated in ambient air at 35°C for 16 to 20 hours. The Mueller-Hinton agar plates without antibiotics served as positive control of bacterial growth. The MIC of each antimicrobial agent was defined as the lowest concentration that inhibited visible growth of the organism. The results were interpreted according to the CLSI guidelines. Escherichia coli ATCC 25922 was included in each set of tests as a control strain [11], [12]. 

#### Statistical analysis

The results were analyzed using descriptive statistics in SPSS software for Windows (version 23 SPSS Inc., Chicago, IL, USA).

## 3 Results

In the present study, 219 Enterobacteriaceae were isolated from the urine specimens of 79 male (36.1%) and 140 female (63.9%) patients. The mean age of patients was 50±22 years. The patients were hospitalized in internal (135, 61.6%), ICU (22, 10%), surgery (47, 21.5%), and pediatric (15, 6.8%) wards. The predominant bacteria isolated were *Escherichia coli* (177 isolates, 80.8%) followed by *Klebsiella pneumonia* (28 isolates, 12.8%), *Enterobacter cloacae* (7 isolates, 3.2%), *Proteus mirabilis*, *Morganella morganii* (2 isolates, 0.9%), *Citrobacter freundii,* and *Proteus vulgaris* (1 isolate, 0.5%). 

Based on disk diffusion and agar dilution assay, the highest frequencies of resistance were found to be against trimethoprim/sulfamethoxazole (96.8%), ampicillin (86.3%), cephazoline (79.4%), trimethoprim (78.5%), and nalidixic acid (68.5%). Low level resistance was observed against fosfomycin (2.7%) and carbapenems (3.2% for imipenem and meropenem, 4.1% for ertapenem) (Table 1 (Tab. 1)). 

According to the agar dilution assay, the resistance rates to ciprofloxacin, nalidixic acid, and levofloxacin were 66.2%, 68.9%, and 58.5%, respectively. The resistance rates to tobramycin, kanamycin, gentamicin, and amikacin were 47.9%, 39.3%, 27.8%, and 5.5%, respectively. The highest frequencies of resistance were found against trimethoprim/sulfamethoxazole (69.8%), sulfamethoxazole (88.1%), and trimethoprim (78.5%). 

Table 2 (Tab. 2) shows the MIC ranges, MIC_50_ and MIC_90_ for each 10 tested antibiotics. The most sensitive drugs were amikacin and gentamicin. 

## 4 Discussion

Enterobacteriaceae are the most frequent pathogens isolated from UTIs, and *Escherichia coli* is the most commonly isolated bacterium [4], [13]. In agreement with other reports [13], [14], *Escherichia coli* was found to be a predominantly identified pathogen (80.0%). 

Anti-metabolite compounds, including trimethoprim and sulfamethoxazole, alone or in combination with each other, are the most common antibiotics in the treatment of UTIs [15]. In our study, a high level of resistance was observed against trimethoprim/sulfamethoxazole (69.8%), sulfamethoxazole (88.1%) and trimethoprim (78.5%). Other studies also reported high resistance levels to these antibiotics in Iran. Arabi et al. [15] reported 60.4% resistance to trimethoprim/sulfamethoxazole (co-trimoxazole) in *Escherichia coli* isolated from UTIs. Khoshbakht et al. [16] reported 69.7% and 63.6% resistance to trimethoprim/sulfamethoxazole among *Klebsiella* spp. and *Escherichia coli*, respectively. According to the Infectious Diseases Society of America (IDSA) guidelines, trimethoprim/sulfamethoxazole is the recommended antibiotic for treatment of UTIs in centers where the prevalence of resistance is <10–20% [17]. However, trimethoprim/sulfamethoxazole does not seem to be a suitable option for empirical therapy of UTIs in our setting.

If the trimethoprim/sulfamethoxazole resistance rate is higher than 20%, quinolones are the drug of choice for treatment of UTIs [17], [18]. An association between the increase of quinolone usage and a decrease of bacterial susceptibility has been described [19]. Quinolone resistance in Enterobacteriaceae has been reported in different countries [19], [20], [21]. Among Enterobacteriaceae isolates, in the present study, 66.2% of isolates were ciprofloxacin resistant, 68.9% were nalidixic acid resistant and 58.5% levofloxacin resistant. Niranjan et al. [22] reported a 75% resistance rate to ciprofloxacin among uropathogenic *Escherichia coli*. Khawcharoenporn et al. [23] reported a 17% resistance to levofloxacin in Enterobacteriaceae isolated from UTIs. Khoshbakht et al. [16] reported a 26.32% resistance to ciprofloxacin and a 60.53% resistance to nalidixic acid among uropathogenic *Escherichia coli*. Factors that may contribute to this disparity include antibiotic use, selective pressure levels, difference in study time and periods, study inclusion criteria, and different geographic regions [23]. Our results showed a high frequency of resistance to quinolones in our setting. Quinolones should not be administrated empirically unless hospital assessment indicates a susceptibility greater than 90% [23], [24]. Therefore, they are not appropriate for empirical therapy in our centers and their administration should be based on antibiotic susceptibility test results before the initiation of treatment. 

In this study, 47.9% of isolates were resistant to tobramycin, 39.3% to kanamycin, 27.8% to gentamicin, and 5.5% to amikacin. Sedighi et al. [25] reported a 3% resistance to amikacin among *Escherichia coli* isolated from UTIs. The resistance rate to trimethoprim/sulfamethoxazole found by Khoshbakht et al. [16] was as 69.74% and 63.64% in *Escherichia coli* and *Klebsiella* spp., respectively. Monotherapy with aminoglycosides produces comparable efficacy with fluoroquinolones and beta-lactam for the treatment of UTIs. These levels, which are higher than the MIC of most Gram-negative organisms, are still detectable in urine for at least 4 days following the last drug administration [26]. In the present study, the MIC results indicated that aminoglycosides are more effective against Enterobacteriaceae isolates than quinolone and anti-metabolite drugs. Amikacin is more effective than other aminoglycosides against Enterobacteriaceae. Amikacin has a rapid dose-dependent bactericidal effect and is excreted via the kidneys. Its distribution in renal parenchyma is also favorable; thus it is administrated alone for long periods in UTIs, including pyelonephritis. Due to nephrotoxicity in previous years, amikacin administration has been decreased. Now it is generally used as part of combination therapy for certain infections due to synergistic properties with beta-lactams. Combination therapy may lead to synergic effects and reduces the distribution of the resistant population of organisms. It is known that single daily dose aminoglycoside usage reduces their complication and increases the antibacterial effect. In a study carried out by Ipekci et al. [27], nephrotoxicity was only reported in 2.8% of all the patients with a single daily dose of amikacin. 

Results obtained from the disk diffusion assay indicated that fosfomycin and carbapenems are the most sensitive antibiotics against Enterobacteriaceae isolated from UTIs in our setting. Carbapenems are effective drugs for extended-spectrum β-lactamases (ESBLs) producing and multidrug resistant (MDR) enterobacteriaceae. Resistance to this class is increasing due to high administration in different geographic areas. However, in most cases, the frequency of carbapenems resistant to Enterobacteriaceae isolated from UTIs is under 5% [28], [29], [30]. Fosfomycin is a broad-spectrum bactericidal drug effective against most Gram-negative and Gram-positive organisms [31]. Among enterobacteriaceae, fosfomycin is one of the most effective antibiotics due to the unique antibacterial mechanism and the synergic effect with beta-lactam, aminoglycosides, and quinolones [31]. The strength of our study lies in the high number of bacteria studied and analyzed by the dilution method. In the present study, the MIC of some antibiotics, such as beta-lactams and fosfomycin, was not determined and the resistance mechanisms and molecular epidemiology of bacterial isolates were not investigated. We suggest that these subjects, as well as the risk factors of infections caused by resistant isolates, should be investigated in future studies.

## 5 Conclusion

The high frequency of antibiotic resistance shows the importance of antibiotic susceptibility testing and a vital need for applicable surveillance programs. Sulfamethoxazole and trimethoprim are not appropriate for empirical therapy in our settings. Quinolones should be administered according to antibiotic susceptibility patterns. Amikacin is the most effective drug in most cases and is best used in combination with a carbapenem or fosfomycin in complicated cases. 

## Notes

### Acknowledgments

This article was written based on a dataset of a M.Sc. thesis registered at Tabriz University of Medical Sciences, Tabriz, Iran. This project was financially supported by the Immunology Research Center, Tabriz University of Medical Sciences.

### Competing interests

The authors declare that they have no competing interests.

## Figures and Tables

**Table 1:**
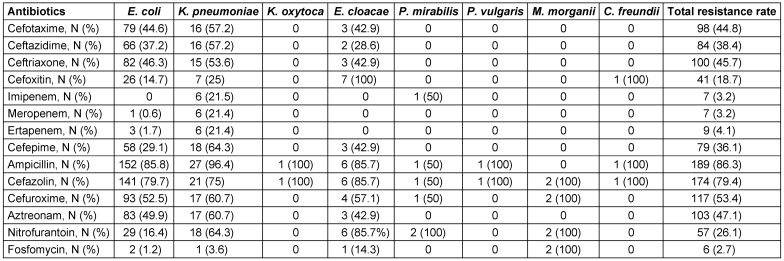
Antibiotic resistance patterns of Enterobacteriaceae isolates according to the disk diffusion assay

**Table 2:**
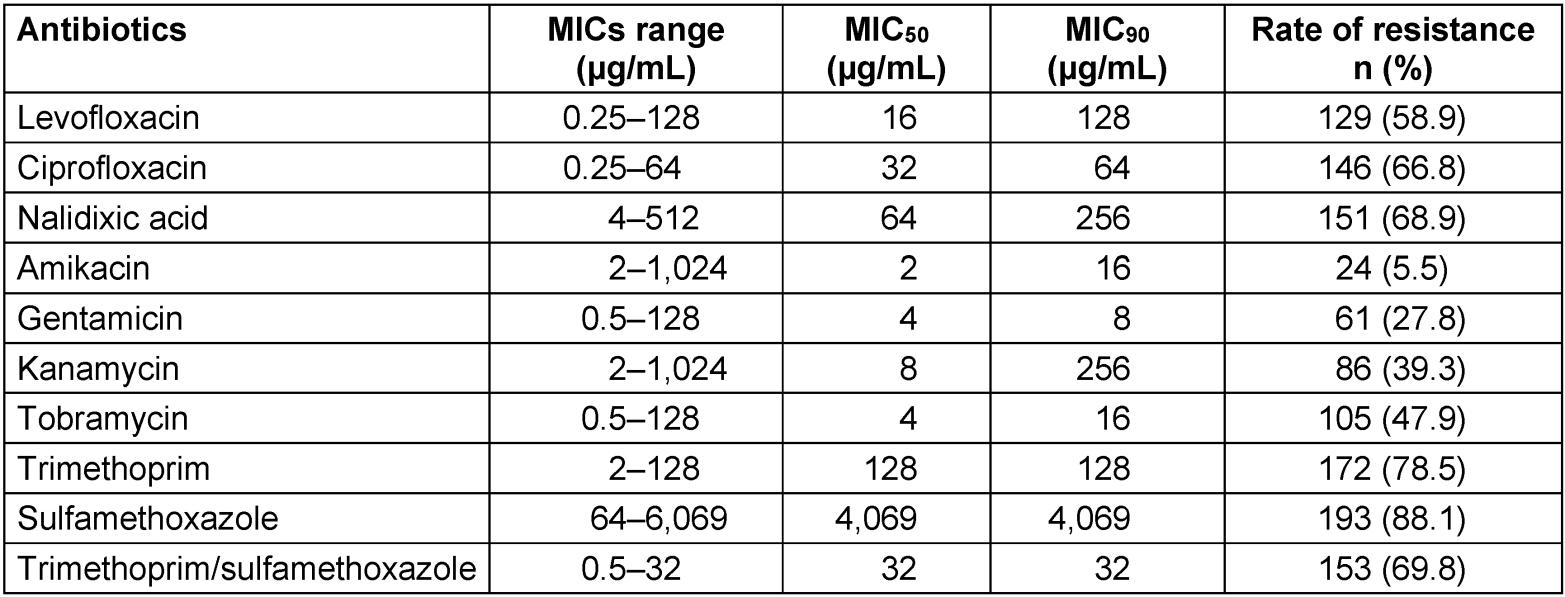
MIC ranges, MIC_50_, and MIC_90_ of antibiotics for Enterobacteriaceae isolates
